# Utility of Extraction-Free SARS-CoV-2 Detection by RT–qPCR for COVID-19 Testing in a Resource-Limited Setting

**DOI:** 10.3390/diseases12090198

**Published:** 2024-08-26

**Authors:** Akua K. Yalley, Selasie Ahiatrogah, Iddrisu I. Moro, Peter Gmagna, Isaac K. Yankson, Anna A. Kafintu-Kwashie, Nicholas I. Nii-Trebi

**Affiliations:** 1 Department of Medical Laboratory Sciences, School of Biomedical and Allied Health Sciences, University of Ghana, Accra P.O. Box KB 143, Ghana; aakafintu-kwashie@ug.edu.gh; 2 Department of Obstetrics and Gynaecology, College of Medicine, Pan African University of Life and Earth Sciences Institute, University of Ibadan, Ibadan P.O. Box 22133, Nigeria; sahiatrogah0373@stu.ui.edu.ng; 3 International Maritime Hospital, Community 3, Tema P.O. Box CO 4297, Ghana; moro.iddi@imah.gov.gh (I.I.M.); peter.gmagna@imah.gov.gh (P.G.); 4 Department of Medical Laboratory Technology, Faculty of Science, Accra Technical University, Barnes Road, Accra P.O. Box GP 561, Ghana; 5 CSIR—Building and Road Research Institute, Kumasi P.O. Box UP40, Ghana; ikyankson@csir.brri.org

**Keywords:** COVID-19, RT–qPCR, qPCR, extraction-free PCR

## Abstract

The COVID-19 epidemic had a profound impact on global health and the economy and Ghana was no exception to its far-reaching consequences. Regarding detection of the causative agent—the severe acute respiratory syndrome coronavirus 2 (SARS-CoV-2), reverse-transcription–qPCR (RT–qPCR) is widely recognized as a very sensitive and reliable diagnostic technique used globally. There are, however, high operational costs in acquiring test kits, equipment, and accessories for RT–qPCR testing, which pose significant challenges in resource-limited settings. Hence, this proof-of-concept study set out to develop a more affordable COVID-19 protocol for use in low or lower-middle-income settings, such as Ghana, that would bypass the traditional extraction process using inexpensive reagents and evaluate the possibility of processing samples collected using wooden shaft swabs. Several less expensive media were used for the extraction-free process. Results demonstrated that direct RT–qPCR assay after 5 min heat inactivation of virus at 95 °C in 0.1× PBS or molecular grade water resulted in viral detection with quantification cycle (Cq) values that are comparable to results obtained following the extraction process. Also, wooden shaft swabs could be used for sampling if incubation times are kept to less than 6 h. The study demonstrates that extraction-free protocols are one way to minimize the cost of COVID-19 testing by RT–qPCR.

## 1. Introduction

The emergence of severe acute respiratory syndrome coronavirus 2 (SARS-CoV-2) occurred in December 2019, marking the identification of a new and distinct coronavirus [1,2]. The virus is responsible for inducing a respiratory ailment known as coronavirus disease 2019 (COVID-19). As of December 2023, the World Health Organization (WHO) reports that COVID-19 has resulted in the loss of over 7 million lives globally [3]. The pandemic it caused had a profound impact on global health and the economy, and Ghana also had its fair share of the far-reaching consequences [4]. Africa’s first case was reported in mid-February and Ghana’s first case was reported on 12 March 2020—a case detected based on polymerase chain reaction (PCR) testing method [5,6]. Since then, over 12 million cases have been diagnosed using PCR and other non-PCR methods [7].

Polymerase chain reaction (PCR) is a useful diagnostic tool for the identification of pathogens and emerging illnesses [8,9]. The PCR technique is highly accurate and sensitive for the detection of nucleic acids and can amplify billions of copies of a specific DNA target from just a single initial copy [9]. Real-time PCR, sometimes referred to as quantitative real-time PCR (qPCR), is a type of PCR in which target nucleic acid product detection can be determined in real time or concurrently with amplification [10]. In the context of detection or relative quantification of a specific RNA molecule, the utilization of qPCR typically involves a series of preparatory steps. First, the total RNA is extracted and purified from the sample. Next, the purified RNA is subjected to a reverse-transcription (RT) reaction, during which complementary DNA (cDNA) is synthesized from the template RNA. The cDNA is then used as the target molecule for the subsequent qPCR reaction [11]. In the context of medical laboratory diagnostics, it is common practice to utilize a single reaction procedure that integrates both the reverse-transcription (RT) and real-time polymerase chain reaction assays [11]. This is often referred to as reverse-transcription quantitative polymerase chain reaction (RT–qPCR) or real-time reverse-transcription-PCR (rRT-PCR).

The provision of SARS-CoV-2 genome sequences during the initial phase of the COVID-19 pandemic, starting from 10 January 2020, played a crucial role in enabling the development of specific primers and established PCR-based laboratory procedures for the detection and identification of the virus [12]. The nucleic acid extraction and amplification by RT–qPCR remains the gold standard for diagnosing SARS-CoV-2 infection [13]. Reports indicate that RT–qPCR demonstrates diagnostic sensitivities ranging from 88% to 99% and specificities ranging from 77% to 100% [14]. The aforementioned assay consequently expedites the conclusive identification of individuals who are asymptomatic, presymptomatic, and symptomatic [15]. Nevertheless, the relative scarcity of testing resources poses a significant challenge in evaluating the ramifications of the COVID-19 pandemic in most African nations, including Ghana—a lower-middle-income country [16]. Furthermore, the current availability of SARS-CoV-2 RT–qPCR testing and testing reagents is constrained in various geographical regions [17]. These may lend credence to the view that the officially reported number of confirmed COVID-19 cases likely underestimates the true extent of the disease.

In fighting infectious diseases, which include COVID-19, the formidable hurdles encountered by low-income and lower-middle-income countries cannot be overlooked [18]. Invariably, challenges facing these countries in accessing basic necessities such as quality health services, electricity, and water [19] tend to hinder the widespread adoption of nucleic acid amplification testing (NAAT), including RT–qPCR. Added to these factors are the scarcity of laboratory scientists proficient in RT–qPCR testing, a shortage of certified biomedical engineers who can effectively calibrate and maintain the laboratory equipment, and the high operational expenses in procuring test kits, equipment, and accessories necessary to undertake RT–qPCR testing. The process of purifying nucleic acids, a preparatory step for RT–qPCR, can also be costly as well as labor-intensive. The labor-intensive aspect, particularly when dealing with large samples, can be addressed using automated methods. However, in resource-limited settings, the cost of using these automated methods can be prohibitive as even the manual extraction kits, which are relatively less expensive, are still considered costly in developing countries such as Ghana. These warrant search for test protocols that are not only affordable, expeditious, and effective but also minimize total reliance on reagents and equipment that have significant cost implications. This is the purpose of this study. The study, conducted in a hospital facility in Ghana, set out to develop a COVID-19 protocol that would bypass the traditional extraction process, using inexpensive reagents and sample collection items. This was a proof-of-concept study.

## 2. Materials and Methods

### 2.1. Samples

A total of 21 archived SARS-CoV-2 positive oropharyngeal swab samples from de-identified COVID-19 patients with Cq values ranging from 17 to 39 were used for this protocol development study. These samples were stored in 0.9% normal saline or 0.1× PBS media at ≥−20 °C. All experimental conditions were done in a minimum of triplicates, as shown in the figure legends in the Section 3. The COVID-19 status of the sample (originally in 0.9% normal saline) used for media and swab testing had been previously confirmed using the traditional manual extraction and One-step RT–qPCR protocol and demonstrated to have Cq values less than 20 (mean of 18). A sample with a low Cq value was needed so we could detect it even after a 1000-fold dilution in different testing media. Samples that were archived in 0.1× PBS and which were used for further testing of 0.1× PBS media conditions were not diluted.

### 2.2. Solutions and Swabs

Consumables tested in this protocol development study included 0.9% *w*/*v* sterile normal saline (Sanbao (GH) pharmaceuticals Ltd., Tema, Ghana); 1× and 0.1× phosphate-buffered saline (PBS) diluted from a 10× PBS stock (70011-036, Gibco Grand Island New York, NY, USA); nuclease-free water (E476-500ml, VWR life science LLC, Solon, OH, USA and 3-07F04-H, Bioconcept, Allschwil, Switzerland); Virotransfer DET/E (CK-VT 17207, CAN KAPTAN Ltd. STI, Istanbul, Turkey), which is an extraction-free viral transport medium; plastic shaft swabs (Bioline, New Delhi, India) and locally manufactured wooden shaft swabs.

### 2.3. Viral RNA Extraction

Viral RNA extraction was done using the quick-RNA viral kit (R1035, Zymo Research, Irvine, CA, USA) with 100 μL of the sample according to the manufacturer’s instructions, where elution was done in 15 μL for standard extraction (Ext) or with some modifications as follows: sample elution volume used was 100 μL (Ext ctrl), which is equivalent to the sample start volume for easier comparison to extraction-free samples. Internal control was not added to the extraction step but rather to the PCR step to allow comparison to the samples in extraction-free media.

### 2.4. Heat Inactivation

Heat inactivation was carried out for 50 μL of samples in 0.9% normal saline, 1× PBS, 0.1× PBS, or nuclease-free water on a heating block set at 95 °C for 5 min. It has been established that heating inactivates the virus, and the heating temperature and time duration were chosen based on a previous study that concluded that using those conditions compared to lower temperatures with prolonged incubation times could effectively inactivate the virus and also result in less RNA fragmentations and thus lower Cq values [11,20]. Subsequent to inactivation, samples were cooled to 4 °C before being subjected to RT–qPCR.

### 2.5. One-Step RT–qPCR

The Allplex 2019-nCoV Assay kit (RP10243X, Seegene, Seoul, Republic of Korea) was used for RT-qPCR according to the manufacturer’s instructions with some modifications as follows: Each PCR reaction contained 2.5 μL of 2019-nCoV MOM reagent mix (containing the primers and probes), 2.5 μL of 5× Real-time One-step buffer, 1 μL of Real-time One-step enzyme, 0.5 μL of exogenous internal control, 3 μL to 4.5 μL of nuclease-free water and 1.5 μL to 3 μL of template, making a total reaction volume of 12.5 μL. The target genes for this assay were the E gene, RDRP gene, N gene, and an exogenous internal control. All assays for this study always included a positive control (Pos ctrl) and a non-template control as a negative control (Neg ctrl). The cycling conditions, according to the manufacturer’s instructions, were as follows: 50 °C for 20 min, 95 °C for 15 min, and 45 cycles of 94 °C for 15 s and 56 °C for 30 s. The magnetic induction cycler (MIC), together with its accompanying software from Biomolecular Systems (Upper Coomera, Australia), was used for the PCR. The target gene was detected if quantification cycle (Cq) values were at or below 40. Experimental conditions were all done in at least triplicates, as shown in the figure legends. For media testing, freezing and thawing tests, and swab shaft-type testing, experimental conditions were done in triplicates. For freezing and thawing post-heating, experimental conditions were done in quadruplicates. For testing across a range of Cq values, the assay was carried out 16 times.

### 2.6. Biosafety Conditions

The study was conducted in a Health Facilities Regulatory Agency (HeFRA)accredited facility for COVID-19 RT–qPCR testing in Ghana. All relevant biosafety measures related to handling suspected SARS-CoV-2 positive samples were adhered to. These include but are not limited to the use of personal protective equipment (PPE), handling samples in a Biosafety Level 2 (BSL-2) hood, and the use of appropriate disinfectants.

### 2.7. Data Analysis

Microsoft Excel was used for data management and data analysis. Continuous data were described as means and standard error of means.

## 3. Results

### 3.1. Evaluating Extraction-Free RT–qPCR Using Samples in Different Media with and without Heat Inactivation

**Figure 1:**
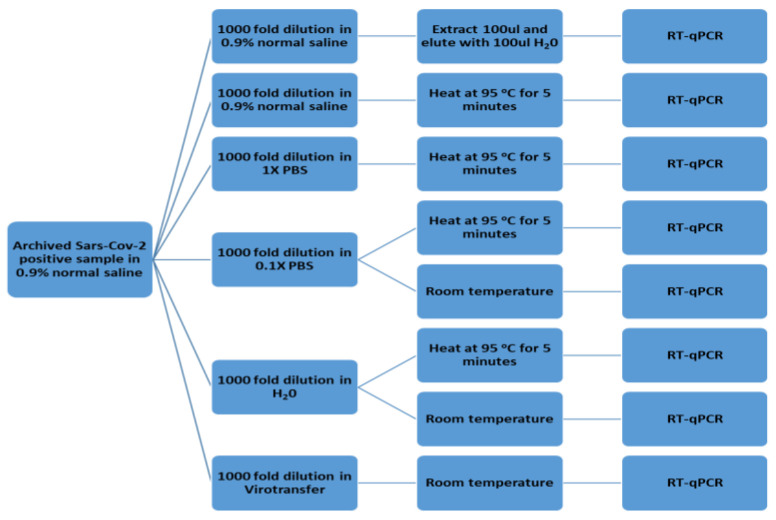
A flow chart showing the procedure for comparing different media conditions in extraction-free SARS-CoV-2 RT–qPCR.

To test which media would be most suitable for COVID-19 RT–qPCR, we first spiked the selected media with a previously confirmed, archived, and de-identified SARS-CoV-2 positive sample to make 1000-fold dilutions of the original concentration (average Cq value of 18) of the sample. This was carried out to ensure there was minimal effect of the original media used for archiving samples on all media to be screened. The media evaluated were as follows: 0.9% normal saline (which was traditionally used in the lab), 1× PBS, 0.1× PBS, nuclease-free water, and Virotransfer DET/E as an extraction-free control buffer. Virotransfer DET/E contains polyethyleneimine-coated tetradecyl dimethyl benzyl ammonium chloride-based nanoparticles (NP), Tris-HCL, Tween-20, Guanidinium isothiocyanate (GITC), and EDTA. This buffer both lyses and preserves the integrity of the released nucleic acids. Thus, samples assayed in Virotranfer DET/E buffer did not need heat inactivation. For a traditional extracted control, 100 μL of the 0.9% normal saline containing the virus was aliquoted and extracted using a viral RNA extraction kit and eluted with 100 μL of nuclease-free water to enable direct comparison between that and the other experimental samples since the other samples would not be concentrated. The extracted control (Ext ctrl) sample was subjected to RT–qPCR together with seven other differently treated samples, namely (1) unextracted samples in 0.9% normal saline, heated at 95 °C for 5 min (0.9% Sal ht), (2) unextracted samples in 1× PBS heated for 5 min at 95 °C (1× PBS ht), (3) unextracted samples in 0.1× PBS, heated for 5 min at 95 °C (0.1× PBS ht), (4) unextracted samples in nuclease-free water, heated for 5 min at 95 °C (H_2_O ht), (5) samples in Virotransfer DET/E (Virotransfer), (6) unextracted samples in 0.1× PBS at room temperature (0.1× PBS rt) and (7) unextracted samples in nuclease-free water at room temperature (H_2_O rt). A flow chart detailing the process is shown in Figure 1. Please note that after heating, all samples were cooled to 4 degrees (including the samples that were not heated) before addition to the RT–qPCR reaction mix.

**Figure 2:**
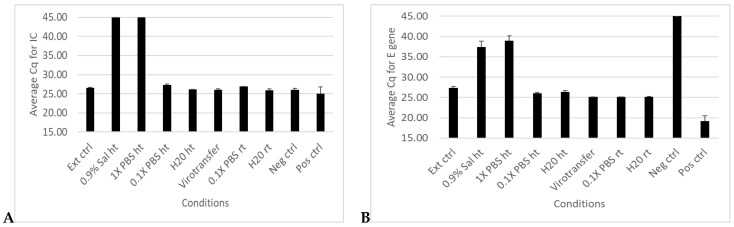

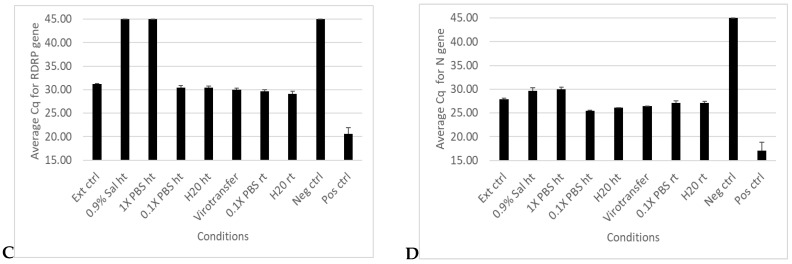
Extraction-free SARS-CoV-2 RT–qPCR results comparing different media conditions. (**A**–**D**) show bar plots of average Cq values of RT–qPCR targeting internal control (IC), E gene, RDRP gene, and N gene, respectively, in a SARS-CoV-2 positive sample in different media conditions. Data represent the mean +/− standard error of the mean for three replicates (*n* = 3).

RT–qPCR results for the internal control (IC) showed detection in all conditions except unextracted samples in 0.9% normal saline and 1× PBS (Figure 2A and Figure S1A). Although the E gene showed detection in all conditions, some inhibition was observed with samples tested in 0.9% normal saline and 1× PBS, which showed average Cq values of 37 and 39 cycles, respectively, compared to Cq value of 27 for the traditionally extracted sample control (Ext ctrl) (Figure 2B and Figure S1B). Results for the RdRP gene showed no detection in the samples tested in 0.9% normal saline and 1× PBS but showed detection in all other conditions (Figure 2C and Figure S1C), while for the N gene, there was amplification in all media conditions tested (Figure 2D and Figure S1D). Notably, nucleic acid detection levels in 0.9% normal saline and 1× PBS were comparable to those in the extracted sample, indicating that those media did not appreciably hinder the detection of the N gene, unlike the other gene targets (Figure 2D and Figure S1D). For all 4 targets, the Cq values for samples in 0.1× PBS (whether heated or at room temperature), in nuclease-free water (whether heated or room temperature), and Virotransfer DET/E were comparable to that of the traditionally extracted sample control (Figure 2 and Figure S1).

In view of the findings above, unextracted samples in 0.9% normal saline and in 1× PBS were eliminated from further testing and analysis since those two conditions appeared not to favor extraction-free RT–qPCR for most of the targets tested. Additionally, tests of samples in 0.1× PBS at room temperature and nuclease-free water at room temperature were also eliminated because although the RT–qPCR results were comparable to tests with heated media, it is practically preferable to use the heated samples, since this condition achieves virus inactivation prior to sample loading for the PCR assay.

### 3.2. Evaluating Degradation of Targets in Extraction-Free RT–qPCR Using Samples in Different Media after Freezing and Thawing

To determine the impact of possible degradation of targets or inhibition of RT–qPCR in the various media due to freeze-thaw actions as compared to what would normally be observed with extracted samples originally incubated in 0.9% normal saline, all samples were frozen at ≥−20 °C for ≥6 days. They were then thawed once and subjected to RT–qPCR after heat inactivation in nuclease-free water or 0.1× PBS. The findings were compared to an extracted control, which had also been subjected to freeze–thawing.

**Figure 3:**
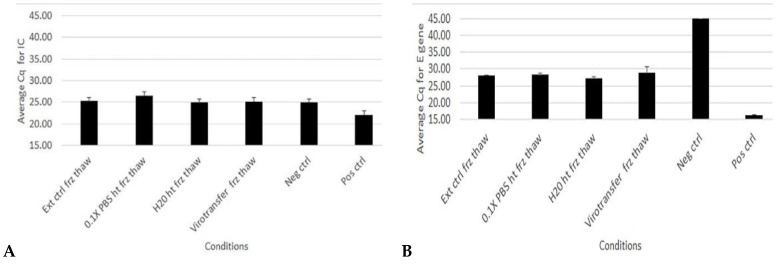

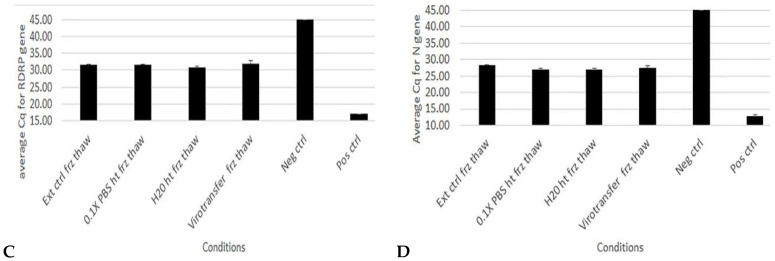
Evaluating degradation of gene targets in extraction-free RT–qPCR using samples in different media after freezing and thawing. (**A**–**D**) show bar plots of average Cq values of RT–qPCR targeting IC, E gene. RDRP gene and N gene, respectively, in a SARS-CoV-2 positive sample subjected to a freezing and thawing cycle. Data represent the mean +/− standard error of the mean for three replicates (n = 3) for all samples except positive and negative controls (*n* = 2).

Results for RT–qPCR targeting internal control (IC), E gene, RDRP, and N genes were comparable for non-extracted frozen and thawed samples and the extracted frozen and thawed samples (Figure 3 and Figure S2).

### 3.3. Evaluating the Impact of Plastic Shaft and Wooden Shaft Swabs in Extraction-Free COVID-19 RT–qPCR Assay after ≤6 h Incubation in Selected Media

We next investigated to what extent, if any, using wooden shaft swabs could impact the RT–qPCR assay as compared to the conventional plastic shaft swabs without the traditional extraction step.

**Figure 4:**
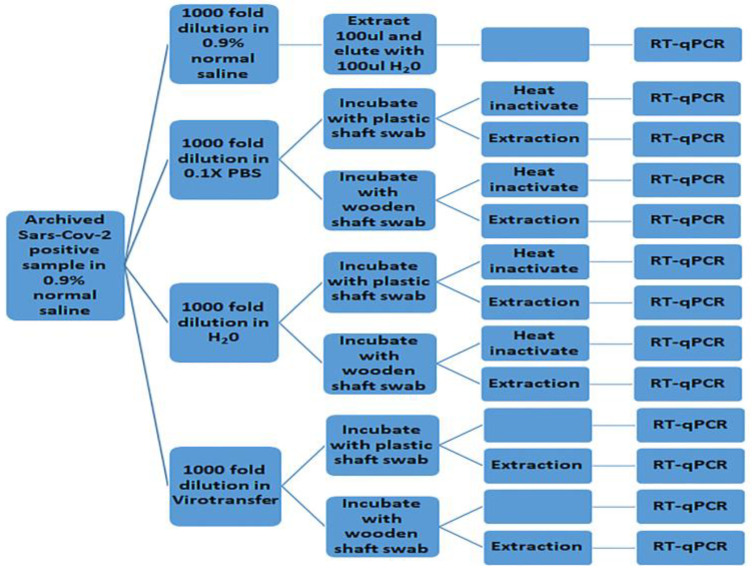
A flow chart showing the process for evaluating wooden shaft swabs in comparison with plastic shaft swabs usage in extraction-free COVID-19 RT–qPCR assay.

The procedure involved first incubating samples with either wooden shaft or plastic shaft swabs for ≤6 h at 4 °C in selected media. Aliquots from 0.1× PBS and nuclease-free water were then heat-inactivated prior to RT–qPCR. See Figure 4 for the flow chart showing the process.

**Figure 5:**
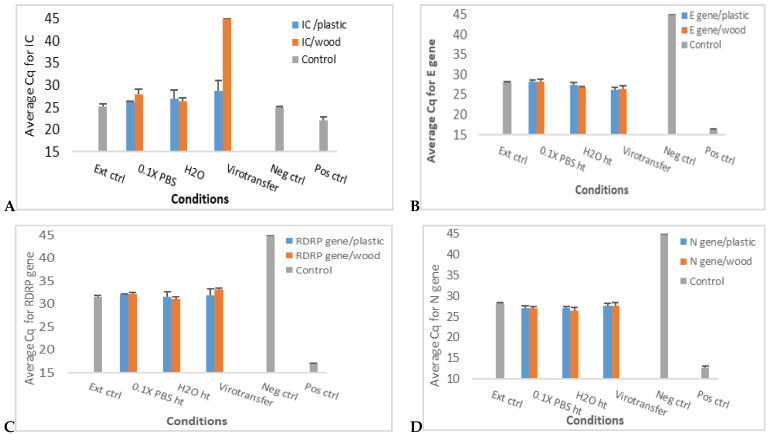
Effects of wooden shaft swabs in comparison with plastic shaft swab usage in extraction-free COVID-19 RT–qPCR assay following ≤6 h incubation in selected media. (**A**–**D**) show bar plots of average Cq values of RT–qPCR targeting IC, E gene, RDRP gene, and N gene, respectively, in a SARS-CoV-2 positive sample subjected to different media conditions after ≤6 h incubation in plastic shaft swabs or wooden shaft swabs. Data represent the mean +/− standard error of the mean for three replicates (n = 3) for all samples except positive and negative controls (*n* = 2).

For the internal control (IC), the Virotransfer DET/E control sample incubated with a wooden shaft swab was the only condition that did not amplify at all (Figure 5A and Figure S3A). All the other conditions were amplified with comparable Cq values (Figure 5A and Figure S3A). For the E, RDRP, and N genes, all conditions were amplified with comparable Cq values (Figure 5B–D and Figure S3B–D).

### 3.4. Evaluating Plastic Shaft Versus Wooden Shaft Swabs in Extraction-Free COVID-19 RT–qPCR Assay after 2-Day Incubation in Selected Media

In evaluating the effect of longer incubation times of swabs in selected media, we incubated the samples with wooden shaft swabs or plastic shaft swabs for 2 days at 4 degrees in 0.1× PBS or nuclease-free water. We then subjected samples to RT–qPCR after heat inactivation. See Figure 4 for the flow chart detailing the process.

**Figure 6:**
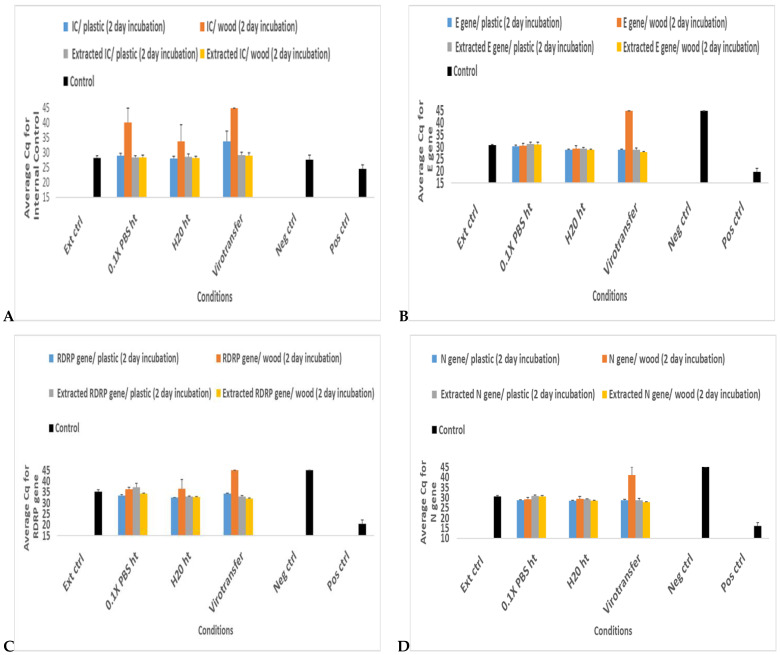
Effects of wooden shaft swabs in comparison with plastic shaft swab usage in extraction-free COVID-19 RT–qPCR assay following 2-day incubation in selected media. (**A**–**D**) show bar plots of average Cq values of RT-qPCR targeting IC, E gene, RDRP gene, and N gene, respectively, in a SARS-CoV-2 positive sample subjected to different media conditions after 2-day incubation in plastic shaft swabs or wooden shaft swabs and also following post-incubation extraction. Data represent the mean +/− standard error of the mean for three replicates (*n* = 3) for all samples except positive and negative controls (*n* = 2).

Except for the N gene that showed inconsistent amplification, for all gene targets, including internal control (IC), the samples in Virotransfer DET/E incubated with the wooden shaft swabs did not amplify (Figure 6 and Figure S4). Amplification of internal control (IC) for samples in 0.1× PBS (0.1× PBS ht) and nuclease-free water (H_2_O ht) both incubated with the wooden shaft swabs were inconsistent as not all replicates amplified as demonstrated in the huge error bars (Figure 6A and Figure S4A). For the RDRP gene, samples in nuclease-free water (H_2_O ht) incubated with wooden shaft swabs had inconsistent amplification as not all replicates amplified, demonstrated in the huge error bar displayed (Figure 6C and Figure S4C). All other parameters for the various gene targets were amplified whether incubated with wooden shaft or plastic shaft swabs (Figure 6 and Figure S4).

To determine if the samples that did not amplify were due to degradation or PCR inhibition, extractions were done on the samples and subjected to RT–qPCR again. For all gene targets, including the internal control, all parameters that either did not amplify or had inconsistent amplification after 2 days of incubation in wooden shaft swabs amplified perfectly (Figure 6 and Figure S4).

### 3.5. Effect of Freeze–Thawing on Extraction-Free COVID-19 RT–qPCR after Heating Samples at 95 °C

**Figure 7:**
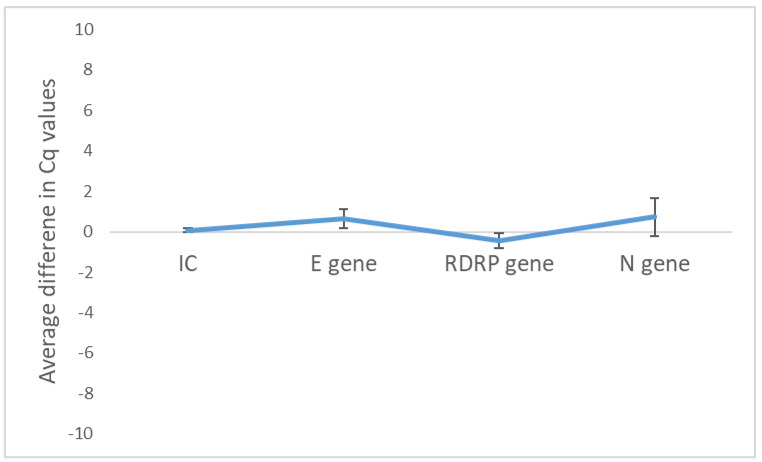
Average difference in Cq values following RT–qPCR after storage of heated samples at −20 °C compared to non-stored heated samples. Data represent the mean +/− standard error of the mean for four replicates (*n* = 4).

To determine what would happen to samples if heated and then frozen at −20 °C to be tested at a later date, four known positive samples archived in 0.1× PBS were either heated and amplified the same day or heated and stored at −20 °C for a minimum of 2 days and then amplified. There was no significant difference in Ct values for all genes, including IC, when heated samples that had been stored at −20 °C for a few days were compared to those that were amplified soon after heating without freeze–thawing (Figure 7)

### 3.6. Extraction-Free COVID-19 RT–qPCR Compared to RT–qPCR Following Standard Extraction of Archived Samples across a Range of Ct Values

**Figure 8:**
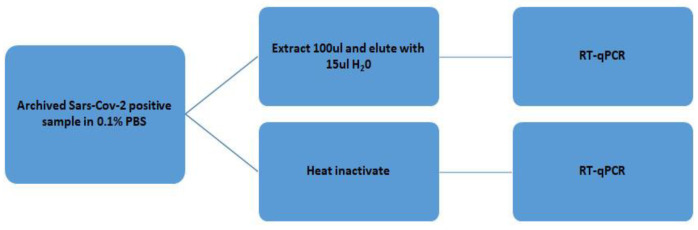
A flow chart showing the process of comparing extraction-free COVID-19 RT–qPCR to RT–qPCR using standard extraction procedures.

We evaluated extraction-free COVID-19 RT–qPCR using 16 archived known positive samples in 0.1× PBS with Cq values that ranged from 25 to 39 when using standard extraction procedures. This was carried out to determine if the detection pattern and sensitivities would differ with different viral load concentrations (as represented by different Cq values), especially as the viral loads approached the limit of detection (Cq values greater than 30). Undiluted Samples were either heat-inactivated or were extracted using a 100 μL sample and eluted with 15 μL DNase/RNase free water, and both conditions were subjected to RT–qPCR. Figure 8 is a flow chart detailing the procedure.

**Figure 9:**
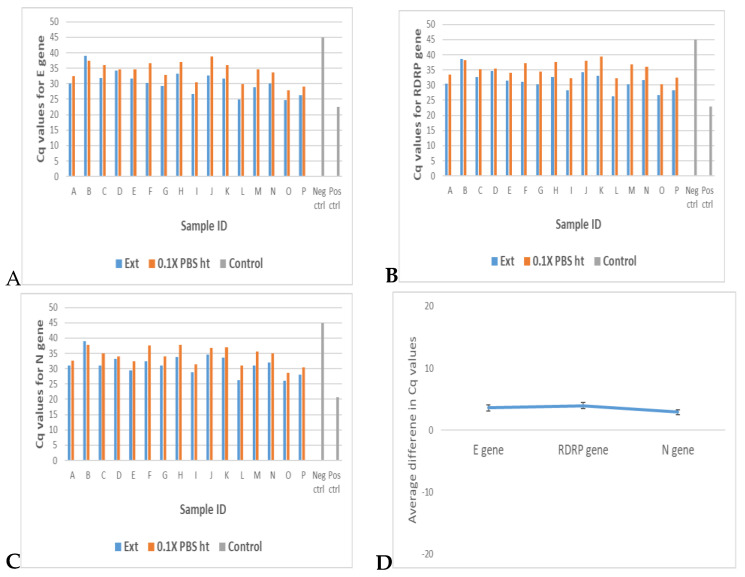
Comparing detection and sensitivities in Extraction-free COVID-19 RT–qPCR versus RT–qPCR following standard extraction of archived samples in 0.1× PBS across a range of Cq values. (**A**–**D**) show bar plots of Cq values of RT-qPCR targeting E gene, RDRP gene, and N gene, respectively, in SARS-CoV-2 positive samples that have undergone standard extraction versus extraction-free samples in 0.1× PBS across a range of Cq values. D shows the average difference in Cq values between extraction-free samples in 0.1× PBS and standard extracted samples. Data represent the mean +/- standard error of the mean for 16 samples (*n* = 16).

Results showed detection in all 16 samples across the range of Ct values for all three target genes (Figure 9A–C and Figure S5). However, in terms of detection sensitivities, the extracted samples were generally slightly ahead of their unextracted counterparts, averaging 3 Cq values lower (Figure 9D and Figure S5).

## 4. Discussion

The high cost of COVID-19 RT–qPCR testing, which traditionally involves RNA extraction, RT reaction, and amplification from samples obtained by swabbing patients with plastic shaft swabs, poses a challenge in widespread dissemination of the test in resource-limited settings such as Ghana. This study explored techniques to perform RT–qPCR on oropharyngeal swab samples without the necessity for RNA extraction and the use of plastic shaft swabs. The findings of this work show that significantly less expensive and less complicated techniques can be used to carry out RT–qPCR-based SARS-CoV-2 testing, obviating the need for RNA extraction kits and accessories.

This study reports that incubating COVID-19 samples at 95 °C for 5 min (to inactivate the virus) in low salt media such as 0.1× PBS or nuclease-free water and subjecting the samples to PCR using commercial kits such as the Allplex 2019-nCoV Assay kit, yields results comparable to those obtainable from extraction. Our results are consistent with other study findings that show the detection of genes using extraction-free PCR protocols [21,22,23,24].

A key focus of this study was the evaluation of each media tested for effectiveness or impact on the PCR assay to determine which extraction-free media would be most suitable for RT–qPCR in the detection of COVID-19. Invariably, the type of media used for incubation is very crucial as some media hinder the PCR reaction. In a similar study, Ngetsa and colleagues reported that 1× PBS did not hinder PCR reaction [25]. The present study, however, observed some limitations with high salt media conditions in the extraction-free detection of certain gene targets. Specifically, except for the N gene on which high salt media appeared to have minimal effect, the remaining gene targets, namely the RDRP gene and the E gene, as well as the internal control, were relatively adversely affected by high salt media. It is possible that the PCR kit Ngetsa and colleagues used, which is different from what this study used, might be more tolerable to high salt media. These findings may underscore a preference for a low salt buffer extraction-free PCR system for enhanced results when using the Allplex 2019-nCoV Assay kit, even though higher salt media may be okay for other kits.

Furthermore, even though wooden shaft swabs are generally less expensive than plastic shaft swabs, it is generally accepted that wooden shaft swabs used for sampling tend to hinder PCR reactions, unlike plastic shaft swabs [26,27,28,29]. This concept was tested in this study to ascertain to what extent sampling with wooden shaft swabs would hinder the extraction-free protocol. It is noteworthy that PCR inhibition was observed in the commercial extraction-free media (one of the controls) containing detergent that had been incubated with wooden shaft swabs, with inhibition worsening for longer incubation times. However, no inhibition was observed for other media when incubation was carried out for less than or equal to 6 h. Also, for the other media, no inhibition was observed for the N gene target even after 2 days of incubation with wooden shaft swabs. This study thus demonstrated that one could use wooden shaft swabs to collect samples in 0.1× PBS and nuclease-free water for COVID-19 RT–qPCR testing, but care should be taken not to leave the swabs in the medium for too long before processing for extraction-free RT–qPCR to avoid inhibition of PCR.

The findings in this study (based on the Allplex 2019-nCoV Assay kit) highlight the N gene as a very robust target for SARS-CoV-2 virus detection. This can either be due to the inherent properties of the primers used or inherent properties in the target itself. This finding is in synchrony with what was shown by Valadan Golchin and others that the N gene was relatively very sensitive for COVID-19 detection, probably due to the abundance of N protein produced in infected cells [30,31]. In a related study in which patients tested positive after two negative SARS-CoV-2 tests (repositivity), the researchers noted the N gene as the main positive gene detected [32]. These and other reports present the N gene as a very reliable gene in general for SARS-CoV-2 detection in view of its high sensitivity and specificity [33].

Samples (either unprocessed or extracted) to be tested for the presence of SARS-CoV-2 are often stored frozen when testing cannot be performed within a few days after samples are received in the lab. It is, however, known that freezing and thawing samples can cause degradation of RNA, which can negatively affect down-stream reactions [34]. The findings in this study showed similar amplification results for extraction-free samples (in 0.1× PBS or nuclease-free water) that had been frozen and thawed when compared to extracted controls that had also been frozen and thawed.

Although the extraction-free method is a useful alternative in resource-limited settings and when one needs quick results, our study shows that standard traditional extraction could still enhance the sensitivity of detection. The one advantage that the standard extraction method has over the extraction-free method is the ability to elute in less volume than the start volume. In addition to making up for potential DNA loss during the extraction process, this concentrates the starting amount of the sample. Concentration increases the relative sensitivity of the PCR, as was seen in Figure 9, compared to when the eluted sample is not concentrated. One way to concentrate a sample in the extraction-free method could be through precipitation methods but these methods are quite laborious and lengthy and may not be a viable option in some circumstances [35]. Another alternative could be to use less transport media volume after sampling. So, for example instead of the up to 3 mL volume of media used for transport and storage after sampling in some labs, the volume can be limited to 0.5 mL with optimization. Individuals with extremely low viral loads and hence high Ct values (greater than 35) using standard methods might be negative using the extraction-free procedure [23,36]. However, it is known that such individuals are those in the recovery state and the test can come out negative sometimes within a day of testing. Also, with such low viral loads, when using the procedure involving nucleic acid extraction, depending on the protocol used, the PCR may not detect target genes. These protocol variations may include volume of transport medium used for sample carriage after initial collection, volume of sample used for extraction and elution and volume of template used for PCR among others [37]. Hence, the protocol described in this study will prove useful especially in resource-limited settings as it helps cut down cost and therefore enables testing a larger number of people with modest resources.

The study had some limitations. The quick-RNA viral kit (Zymo research, R1035) and the Allplex 2019-nCoV Assay kit were the only viral RNA extraction kit and RT–qPCR kit used for this study. Since different commercial PCR kits have different compositions and protocols do vary, the need to optimize protocols as and when different PCR kits are used is imperative. Also, since only oropharyngeal swabs were used for the study, outcomes may be different for other sample types. In addition to the above, a sample size of 21 was low. In the future, more extensive studies will be done, which would include a larger sample size, different sample types, and different kit types used for COVID-19 testing, among others.

## 5. Conclusions

In conclusion, as a proof of concept, we have developed an extraction-free COVID-19 RT–qPCR testing approach that is not only less expensive but less labor-intensive as compared to the manual extraction methods used in laboratories that cannot afford the automated extraction systems. Moreover, this test protocol reduces the turnaround time to obtain results. Adopting similar approaches in various laboratories would make COVID-19 testing more affordable and accessible in resource-limited settings like Ghana.

## Data Availability

Data are contained within the article or Supplementary Material.

## Supplementary Materials

The following supporting information can be downloaded at: https://www.mdpi.com/article/10.3390/diseases12090198/s1, Figure S1: Sample amplification plots of extraction-free SARS-CoV-2 RT–qPCR results comparing different buffer conditions; Figure S2: Sample amplification plots of evaluating degradation of gene targets in extraction-free RT–qPCR using samples in different media after freezing and thawing; Figure S3: Sample amplification plots of effects of wooden shaft swabs in comparison with plastic shaft swabs usage in extraction-free COVID-19 RT–qPCR assay following ≤6 h incubation in selected media; Figure S4: Sample amplification plots of effects of wooden shaft swabs in comparison with plastic shaft swabs usage in extraction-free COVID-19 RT–qPCR assay following 2 day incubation in selected media. Figure S5: Sample amplification plots for evaluating detection and sensitivities in extraction-free COVID-19 RT–qPCR versus RT–qPCR following standard extraction of archived samples in 0.1× PBS across a range of Cq values.

## Abbreviations

SARS-CoV-2	severe acute respiratory syndrome coronavirus 2
RT–qPCR	reverse-transcription quantitative polymerase chain reaction
rRT-PCR	real-time reverse-transcription-PCR
Cq	quantification cycle
COVID-19	coronavirus disease 2019
WHO	World Health Organization
PCR	polymerase chain reaction
qPCR	quantitative real-time PCR
RT	reverse-transcription
cDNA	complementary DNA
Pos ctrl	positive control
Neg ctrl	Negative control
MIC	magnetic induction cycler
GITC	Guanidinium isothiocyanate
Ext ctrl	extracted control
0.9% Sal ht	unextracted samples in 0.9% normal saline, heated at 95 °C for 5 min
1× PBS ht	unextracted samples in 1× PBS heated for 5 min at 95 °C
0.1× PBS ht	unextracted samples in 0.1× PBS, heated for 5 min at 95 °C
H_2_O ht	unextracted samples in nuclease-free water, heated for 5 min at 95 °C
0.1× PBS rt	unextracted samples in 0.1× PBS at room temperature
H_2_O rt	unextracted samples in nuclease-free water at room temperature
Frz	Freeze
Ext	Extracted
HeFRA	Health Facilities Regulatory Agency
PPE	personal protective equipment
BSL-2	Biosafety Level 2

## References

[1] Wu F., Zhao S., Yu B., Chen Y.-M., Wang W., Song Z.-G., Hu Y., Tao Z.-W., Tian J.-H., Pei Y.-Y. (2020). A new coronavirus associated with human respiratory disease in China. Nature.

[2] Zhu N., Zhang D., Wang W., Li X., Yang B., Song J., Zhao X., Huang B., Shi W., Lu R. (2020). A novel coronavirus from patients with pneumonia in China, 2019. N. Engl. J. Med..

[3] WHO World Health Organization. WHO Coronavirus (COVID-19) Dashboard 2023. https://covid19.who.int/.

[4] Yalley A.K., Ahiatrogah S., Yalley A.B., Yankson I.K., Nii-Trebi N.I., Yalley A.A. (2023). Did Ghana Do Enough? A Scientometric Analysis of COVID-19 Research Output from Ghana within the African Context. Diseases.

[5] Kenu E., Frimpong J., Koram K. (2020). Responding to the COVID-19 pandemic in Ghana. Ghana. Med. J..

[6] AfricaCDC Africa Identifies First Case of Coronavirus Disease: Statement by the Director of Africa CDC: 15th February 2020. https://africacdc.org/news-item/africa-identifies-first-case-of-coronavirus-disease-statement-by-the-director-of-africa-cdc/.

[7] Worldometer. https://www.worldometers.info/coronavirus/.

[8] Ksiazek T.G., Erdman D., Goldsmith C.S., Zaki S.R., Peret T., Emery S., Tong S., Urbani C., Comer J.A., Lim W. (2003). A novel coronavirus associated with severe acute respiratory syndrome. N. Engl. J. Med..

[9] Yang S., Rothman R.E. (2004). PCR-based diagnostics for infectious diseases: Uses, limitations, and future applications in acute-care settings. Lancet Infect. Dis..

[10] Maddocks S., Jenkins R. (2017). Chapter 4-Quantitative PCR: Things to consider. Understanding PCR.

[11] Smyrlaki I., Ekman M., Lentini A., Rufino de Sousa N., Papanicolaou N., Vondracek M., Aarum J., Safari H., Muradrasoli S., Rothfuchs A.G. (2020). Massive and rapid COVID-19 testing is feasible by extraction-free SARS-CoV-2 RT-PCR. Nat. Commun..

[12] Abdullahi I.N., Emeribe A.U., Akande A.O., Ghamba P.E., Adekola H.A., Ibrahim Y., Dangana A. (2020). Roles and challenges of coordinated public health laboratory response against COVID-19 pandemic in Africa. J. Infect. Dev. Ctries..

[13] WHO: World Health Organization (2020). Laboratory Testing for Coronavirus Disease (COVID-19) in Suspected Human Cases.

[14] Castro R., Luz P.M., Wakimoto M.D., Veloso V.G., Grinsztejn B., Perazzo H. (2020). COVID-19: A meta-analysis of diagnostic test accuracy of commercial assays registered in Brazil. Braz. J. Infect. Dis..

[15] Okba N.M., Müller M.A., Li W., Wang C., GeurtsvanKessel C.H., Corman V.M., Lamers M.M., Sikkema R.S., de Bruin E., Chandler F.D. (2020). Severe acute respiratory syndrome coronavirus 2−specific antibody responses in coronavirus disease patients. Emerg. Infect. Dis..

[16] Worldbank World Bank 2023 World Bank Country and Lending Groups. https://www.worldbank.org/en/home/.

[17] Hoffman T., Nissen K., Krambrich J., Rönnberg B., Akaberi D., Esmaeilzadeh M., Salaneck E., Lindahl J., Lundkvist Å. (2020). Evaluation of a COVID-19 IgM and IgG rapid test; an efficient tool for assessment of past exposure to SARS-CoV-2. Infect. Ecol. Epidemiol..

[18] Yalley A.K., Ahiatrogah S., Kafintu-Kwashie A.A., Amegatcher G., Prah D., Botwe A.K., Adusei-Poku M.A., Obodai E., Nii-Trebi N.I. (2022). A systematic review on suitability of molecular techniques for diagnosis and research into infectious diseases of concern in resource-limited settings. Curr. Issues Mol. Biol..

[19] Sharma M.G., Popli H. (2023). Challenges for Lower-Middle-Income Countries in Achieving Universal Healthcare: An Indian Perspective. Cureus.

[20] Chin A.W., Chu J.T., Perera M.R., Hui K.P., Yen H.-L., Chan M.C., Peiris M., Poon L.L. (2020). Stability of SARS-CoV-2 in different environmental conditions. Lancet Microbe.

[21] Morecchiato F., Coppi M., Baccani I., Maggini N., Ciccone N., Antonelli A., Rossolini G.M. (2021). Evaluation of extraction-free RT-PCR methods for faster and cheaper detection of SARS-CoV-2 using two commercial systems. Int. J. Infect. Dis..

[22] Chu A.W.-H., Chan W.-M., Ip J.D., Yip C.C.-Y., Chan J.F.-W., Yuen K.-Y., To K.K.-W. (2020). Evaluation of simple nucleic acid extraction methods for the detection of SARS-CoV-2 in nasopharyngeal and saliva specimens during global shortage of extraction kits. J. Clin. Virol..

[23] Kim Y.K., Chang S.H. (2021). Clinical usefulness of extraction-free PCR assay to detect SARS-CoV-2. J. Virol. Methods.

[24] Jayaprakasam M., Aggarwal S., Mane A., Saxena V., Rao A., Bandopadhyay B., Chakraborty B., Guha S.K., Taraphdar M., Acharya A. (2021). RNA-extraction-free diagnostic method to detect SARS-CoV-2: An assessment from two states, India. Epidemiol. Infect..

[25] Ngetsa C., Osoti V., Okanda D., Marura F., Shah K., Karanja H., Mugo D., Gitonga J., Mutunga M., Lewa C. (2023). Validation of saline, PBS and a locally produced VTM at varying storage conditions to detect the SARS-CoV-2 virus by qRT-PCR. PLoS ONE.

[26] CDC: Centre for Disease Control and Prevention. 2022. Interim Guidelines for Collecting, Handling, and Testing Clinical Specimens from Persons for Coronavirus Disease 2019 (COVID-19) URL. https://www.cdc.gov/coronavirus/2019-ncov/lab/guidelines-clinical-specimens.html.

[27] Hedman J., Knutsson R., Ansell R., Rådström P., Rasmusson B. (2013). Pre-PCR processing in bioterrorism preparedness: Improved diagnostic capabilities for laboratory response networks. Biosecurity Bioterrorism Biodefense Strategy Pract. Sci..

[28] Lee A., Cooper T. (1995). Improved direct PCR screen for bacterial colonies: Wooden toothpicks inhibit PCR amplification. Biotechniques.

[29] Nairz M., Bellmann-Weiler R., Ladstaetter M., Schuellner F., Zimmermann M., Koller A.-M., Blunder S., Naschberger H., Klotz W., Herold M. (2021). Overcoming limitations in the availability of swabs systems used for SARS-CoV-2 laboratory diagnostics. Sci. Rep..

[30] Valadan R., Golchin S., Alizadeh-Navaei R., Haghshenas M., Zargari M., Mousavi T., Ghamati M. (2022). Differential gene expression analysis of common target genes for the detection of SARS-CoV-2 using real time-PCR. AMB Express.

[31] Vogels C.B., Brito A.F., Wyllie A.L., Fauver J.R., Ott I.M., Kalinich C.C., Petrone M.E., Casanovas-Massana A., Catherine Muenker M., Moore A.J. (2020). Analytical sensitivity and efficiency comparisons of SARS-CoV-2 RT–qPCR primer–probe sets. Nat. Microbiol..

[32] Zhang X., Li M., Zhang B., Chen T., Lv D., Xia P., Sun Z., Shentu X., Chen H., Li L. (2020). The N gene of SARS-CoV-2 was the main positive component in repositive samples from a cohort of COVID-19 patients in Wuhan, China. Clin. Chim. Acta.

[33] Abbasi H., Tabaraei A., Hosseini S.M., Khosravi A., Nikoo H.R. (2022). Real-time PCR Ct value in SARS-CoV-2 detection: RdRp or N gene?. Infection.

[34] Kellman B.P., Baghdassarian H.M., Pramparo T., Shamie I., Gazestani V., Begzati A., Li S., Nalabolu S., Murray S., Lopez L. (2021). Multiple freeze-thaw cycles lead to a loss of consistency in poly (A)-enriched RNA sequencing. BMC Genom..

[35] Delgado-Diaz D.J., Sakthivel D., Nguyen H.H., Farrokzhad K., Hopper W., Narh C.A., Richards J.S. (2022). Strategies that facilitate extraction-free SARS-CoV-2 nucleic acid amplification tests. Viruses.

[36] Barza R., Patel P., Sabatini L., Singh K. (2020). Use of a simplified sample processing step without RNA extraction for direct SARS-CoV-2 RT-PCR detection. J. Clin. Virol..

[37] Rhoads D.D., Pinsky B.A. (2022). The truth about SARS-CoV-2 cycle threshold values is rarely pure and never simple. Clin. Chem..

