# Supplementary material for: Utility of Extraction-Free SARS-CoV-2 Detection by RT–qPCR for COVID-19 Testing in a Resource-Limited Setting
Source: Diseases. 2024 Aug 26;12(9):198. doi: 10.3390/diseases12090198 (PMC11430816; doi:10.3390/diseases12090198)
Supplement: Supplementary file 1 [file diseases-12-00198-s001.zip › diseases-3097668-supplementary.pdf]

Utility of Extraction-Free SARS-CoV-2 Detection by RT-qPCR for COVID-19 Testing in a Resource Limited Setting

Supplementary Figures

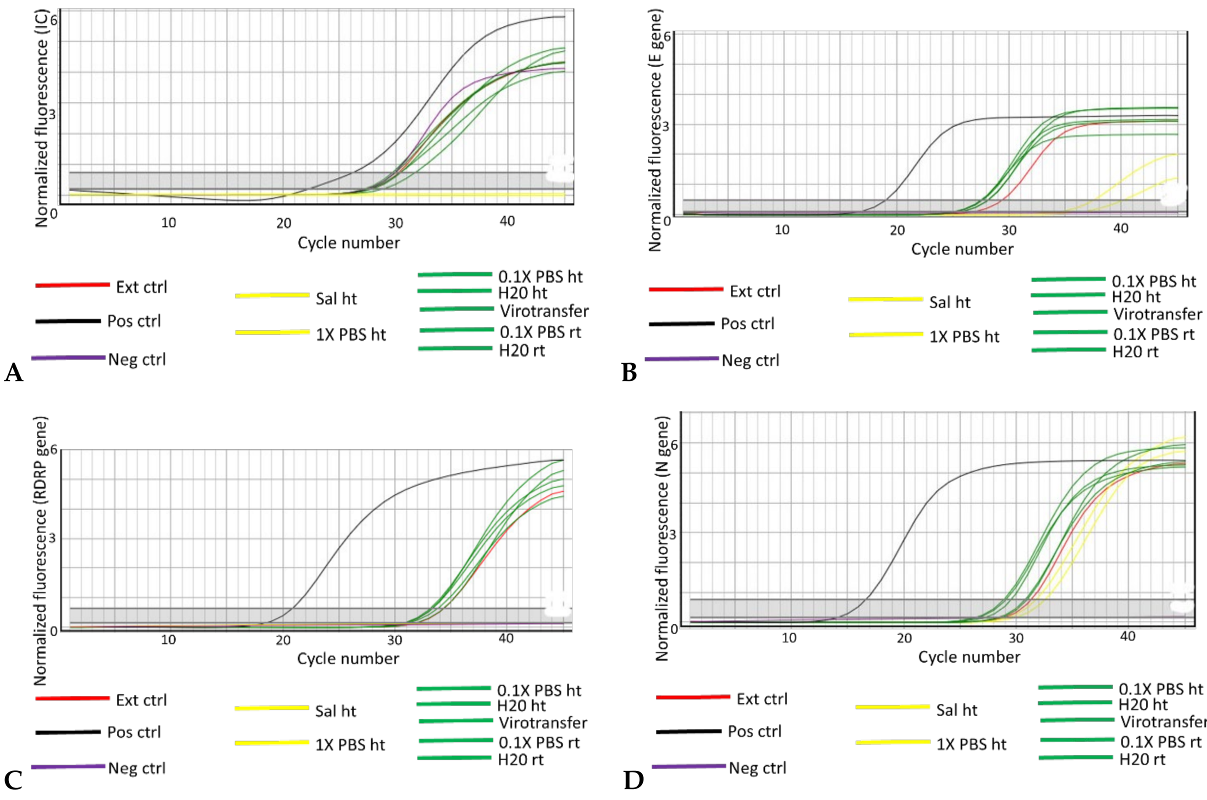

Figure S1. Sample amplification plots of extraction free SARS-CoV-2 RT-qPCR results comparing different buffer conditions. A, B, C and D display amplification plots of normalized fluorescent reporter values as a function of RT-qPCR cycle targeting internal control (IC), E gene, RDRP gene and N gene respectively in a SARS-CoV-2 positive sample in different buffer conditions.

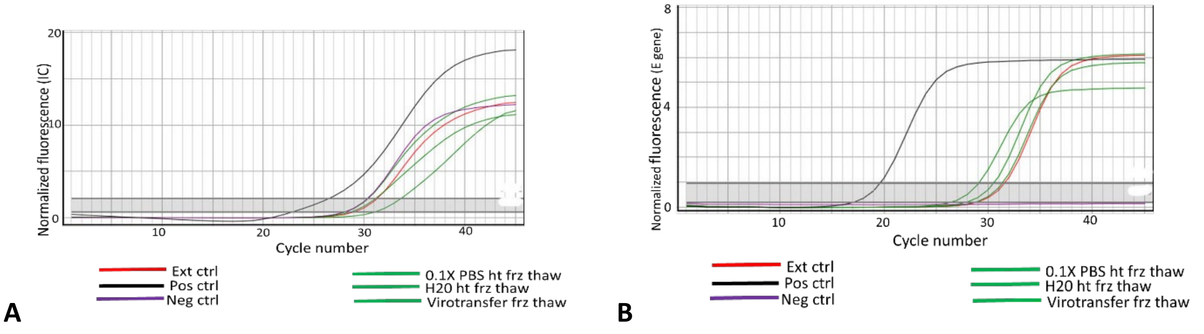

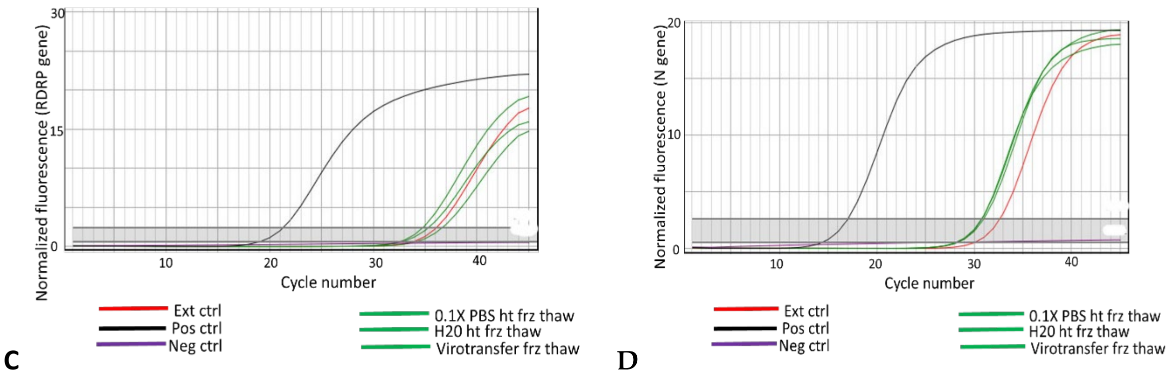

Figure S2. Sample amplification plots of evaluating degradation of gene targets in extraction free RT-qPCR using samples in different media after freezing and thawing. A, B, C and D display amplification plots of normalized fluorescent reporter values as a function of RT-qPCR cycle; targeting internal control (IC), E gene. RDRP gene and N gene respectively in a SARS-CoV-2 positive sample subjected to a freezing and thawing cycle**.**

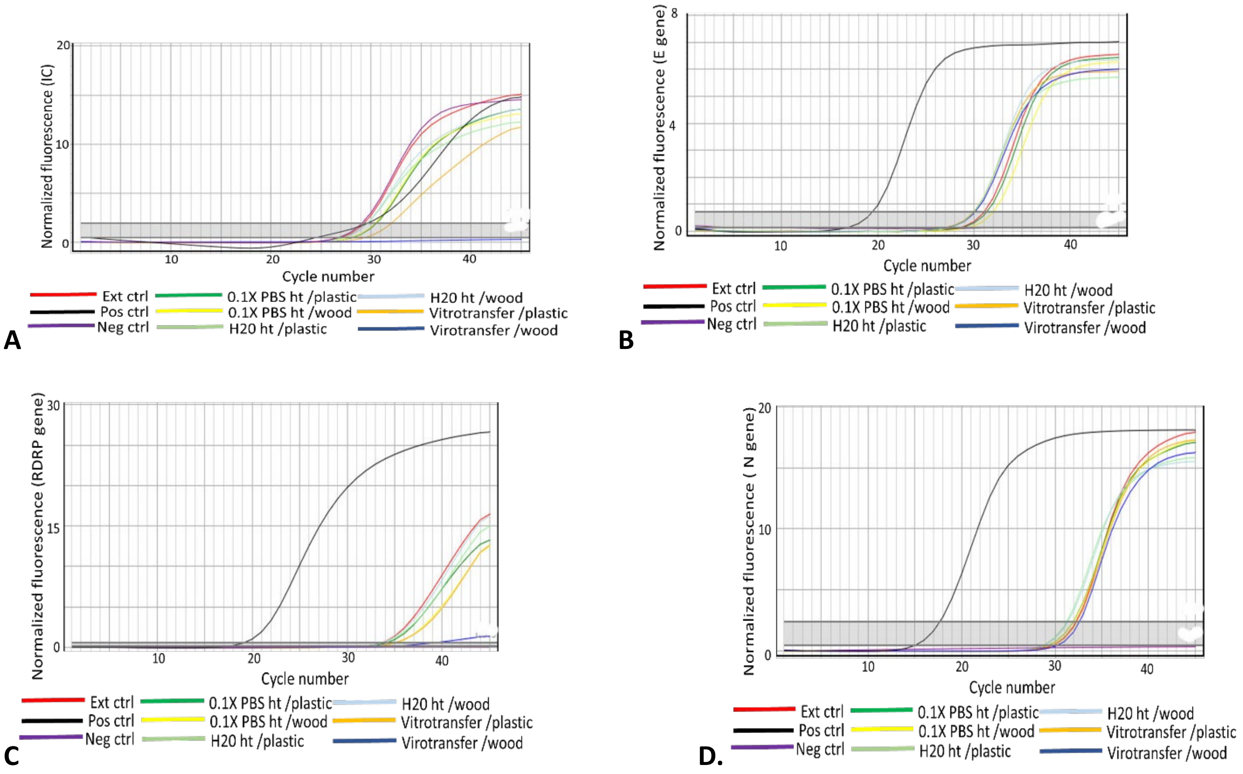

Figure S3: Sample amplification plots of effects of wooden shaft swabs in comparison with plastic shaft swabs usage in extraction free COVID-19 RT-qPCR assay following $\leq$ 6 hour incubation in selected media. A, B, C and D display amplification plots of normalized fluorescent reporter values as a function of qPCR cycle targeting IC, E gene, RDRP gene and N gene respectively in a SARS-CoV-2 positive sample

subjected to different media conditions after < 6 hour incubation in plastic shaft swabs or wooden shaft swabs.

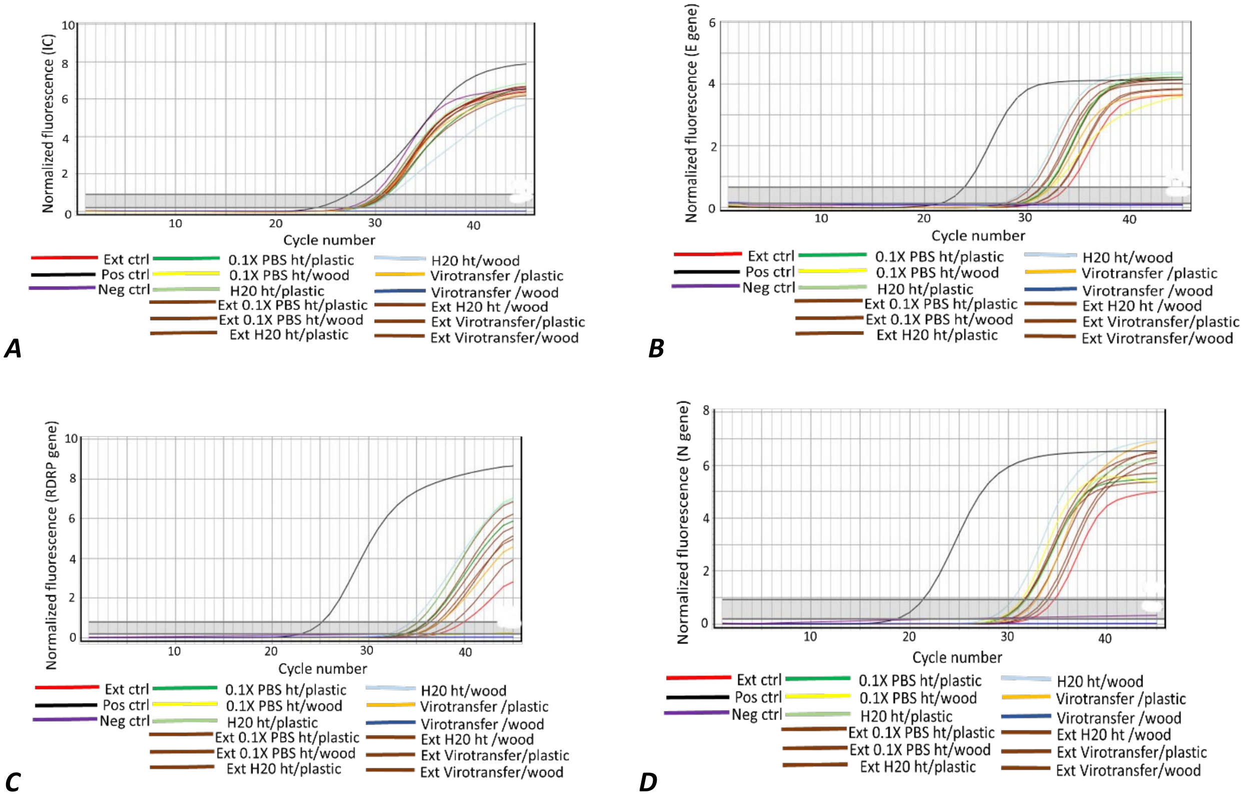

Figure S4. Sample amplification plots of effects of wooden shaft swabs in comparison with plastic shaft swabs usage in extraction free COVID-19 RT-qPCR assay following <u>2 day</u> incubation in selected media. *A, B, C and D display amplification plots of normalized fluorescent reporter values as a function of qPCR cycle targeting IC, E, RDRP, and N genes respectively in a SARS-CoV-2 positive sample subjected to different media conditions after 2 day incubation in plastic shaft swabs or wooden shaft swabs; and also following post incubation extraction.*

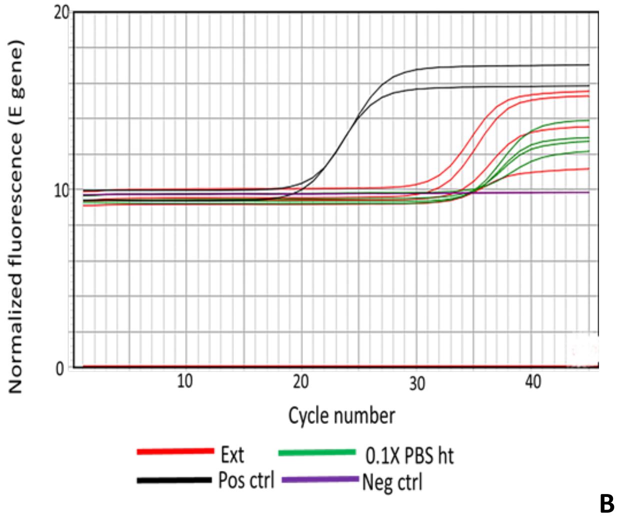

A

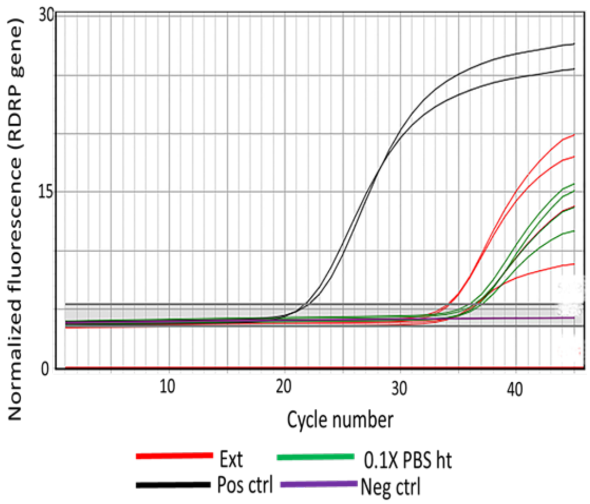

B

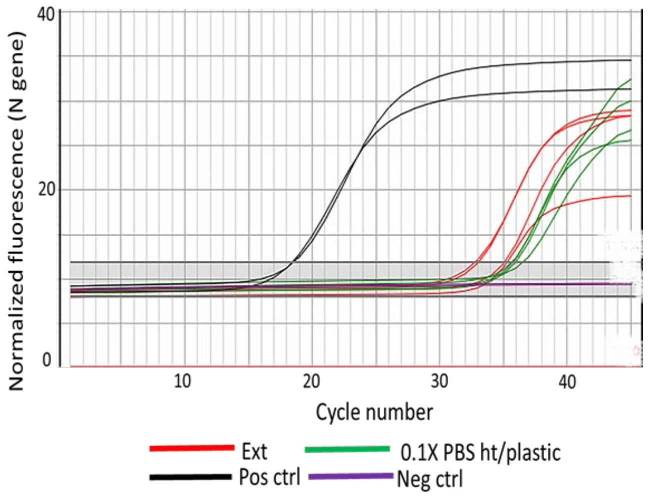

C

***Figure S5. Sample amplification plots for evaluating* detection and sensitivities in extraction free COVID-19 RT-qPCR versus RT-qPCR following standard extraction of archived samples in 0.1XPBS across a range of Cq values.** *A, B and C display amplification plots of normalized fluorescent reporter values as a function of RT- qPCR cycle targeting E, RDRP, and N genes respectively in SARS-CoV-2 archived positive samples (with a range of Cq values) subjected to standard extraction compared to their extraction free counterparts.*
